# Persistence and Risk Assessment of Biofilm-Forming MDR and XDR Bacteria on Non-Poultry Meat Contact Surfaces in Wah Cantt, Pakistan

**DOI:** 10.3390/microorganisms14051051

**Published:** 2026-05-07

**Authors:** Lubna Shakoor, Shumaila Naz, Anas Rashid, Muhammad Idrees

**Affiliations:** 1 Department of Biosciences, University of Wah, Quaid Campus, Wah 47040, Pakistan; lubna.mrl@gmail.com (L.S.); muhammad.idrees@uow.edu.pk (M.I.); 2 Saint Camillus International University of Health and Medical Sciences, Via di Sant’Alessandro 8, 00131 Rome, Italy

**Keywords:** antimicrobial resistance, biofilm, extensively drug-resistant, one health, food safety, public health risk, WHO AWaRe framework

## Abstract

Biofilms on meat-contact surfaces pose critical food safety risks. This study investigates the interplay between biofilm architecture, metabolic vigor, and antimicrobial resistance on retail surfaces in Pakistan. Screening 300 isolates from 120 surfaces identified 42 high-risk biofilm formers. Comprehensive phenotypic screening revealed that standard visual assays severely underestimate the viability of environmental strains. Biofilm biomass and metabolic activity correlated positively (Spearman’s ρ = 0.656, *p* < 0.001). Crucially, Ordinary Least Squares regression established that metabolic vigor, rather than physical biomass, independently predicts resistance severity. Phenotypic profiling revealed a high-risk landscape with 81.8% multidrug-resistant and 18.2% extensively drug-resistant isolates, including resistance to colistin and Linezolid. Alarmingly, 79.5% of critical resistance phenotypes compromised WHO Reserve category antibiotics, escalating to 100% on mincer machines. Ecological analysis demonstrated surface-driven partitioning; porous wood boards fostered diverse *Enterobacteriaceae*, while mincers selected for uniformly resistant clades. These findings highlight processing machinery as resilient reservoirs for untreatable pathogens, necessitating targeted anti-biofilm measures, such as matrix-degrading enzymes. Bridging a critical knowledge gap, this study is among the earliest integrated ecological analyses combining phylogenetic, metabolic, and resistance profiling in Pakistan’s non-poultry meat sector.

## 1. Impact Statement/Summary

This study represents a vital integrated analysis of phylogenetic and metabolic profiles in Pakistan’s critically underexplored non-poultry sector. This research establishes that while biofilm biomass strongly correlates with high metabolic activity, it is mathematically proven that a pathogen’s metabolic vigor—rather than the sheer physical thickness of the biofilm—is what drives the persistence and severity of multidrug-resistant pathogens. By identifying mincer machines and cutting boards as metabolic hotspots where physical microtopography and mechanical stress actively select for strains resistant to last-resort antibiotics such as Colistin and Linezolid. Crucially, mapping these profiles against the global WHO AWaRe framework reveals that 79.5% of critical resistance phenotypes compromise ‘Reserve’ category therapeutics, a severe public health threat that escalates to an alarming 100% on mechanical mincers. These findings highlight a critical failure in current food-safety sanitation protocols. Furthermore, we identify a critical methodological flaw: standard visual assays fail to detect these hyper-viable environmental threats, underscoring the absolute necessity of quantitative biosecurity surveillance. Ultimately, this work necessitates a targeted shift toward ecology-based, biofilm-targeted interventions, such as matrix-degrading enzymes and lytic bacteriophages, to mitigate the dissemination of extensively drug-resistant clades and disrupt transmission networks through the food supply chain.

## 2. Introduction

Biofilms are complex assemblies of bacterial cells attached to surfaces and enclosed within an extracellular material produced by the bacteria themselves. Such biofilms are common in various environments, including food processing facilities, where their existence poses serious problems to food safety. The biofilm matrix primarily consists of exopolysaccharides, proteins, and nucleic acids that coat bacterial cells, providing a protective layer that enhances survival under environmental stress and in the presence of antimicrobial agents. It increases the persistence of pathogens that depend on biofilm formation, making them difficult to remove from food-contact surfaces. The complex microbial colonies attach to surfaces and form an extracellular matrix, conferring resistance to cleaning solutions and antibiotics [1]. These biofilms are commonly found in meat retail shops on equipment such as knives, cutting boards, and mincing machines, where they act as reservoirs for pathogenic bacteria [2]. Biofilm formation is recognized as a primary factor contributing to the survival of antimicrobial-resistant bacteria in food chains, particularly in low-resource settings where sanitation is inadequate [3]. In food retail markets, contact surfaces (i.e., meat) have been found to harbor resistant isolates at alarming levels, particularly in developing countries where antibiotic use in livestock is generally unregulated [4]. Researchers have raised concerns that the overuse of food-grade disinfectants in these locations may accelerate the global development of antimicrobial-resistant bacteria due to heightened cross-resistance and the emergence of resistant, multi-purpose, multifunctional biofilms that contribute to antimicrobial resistance [5].

Antimicrobial resistance (AMR) in low- and middle-income countries (LMICs) has shown an alarming upward trend in food-animal populations over the last two decades [6]. This escalating threat necessitates a multi-sectoral response as outlined in the One Health Joint Plan of Action (2022–2026), which emphasizes the interconnectedness of human, animal, and environmental health [7]. In this context, veterinary medicine plays a pivotal role in mitigating the spillover of resistant pathogens into the food supply chain [8]. Multidrug-resistant (MDR) and extensively drug-resistant (XDR) bacteria pose a serious challenge to the global meat industry, and a recent study has reported increasing resistance in foodborne pathogens such as *Escherichia coli* and *Salmonella* spp. [9]. The meat industry in Pakistan, which relies heavily on informal retail markets, presents specific risks for AMR spread through contaminated equipment and handling practices [10]. Although this represents a significant public health hazard, insufficient systematic research has been conducted on biofilm-related AMR in Pakistan’s non-poultry meat retail sector. Numerous studies have investigated biofilm-forming bacteria in poultry and other food systems [11]. These high-risk environments are particularly evident in beef and mutton shops, where the same equipment is frequently reused, and sterilization procedures are often inadequately performed [12]. Wooden cutting boards are highly porous, and the cutting machines are difficult to clean, thereby creating ideal conditions for biofilm formation and bacterial persistence. These risks are further exacerbated by the routine use of disinfectants, which may unintentionally select resistant isolates [5].

Biofilm development plays a critical role in bacterial survival and virulence. Bacteria in biofilm form are significantly more resistant to environmental stressors, disinfectants, and antibiotics than planktonic counterparts [13]. With the increasing prevalence of antibiotic resistance among foodborne pathogens, understanding the dynamics of resistance and biofilm formation on meat-contact surfaces has become essential. Biofilm-forming bacteria contaminating meat-processing surfaces pose a substantial threat to food safety and public health, as biofilms enhance bacterial persistence, facilitate horizontal gene transfer, and promote increased tolerance to disinfectants and antimicrobial agents. Hygiene practices in informal meat markets in Pakistan often fall short of international standards, creating conditions conducive to the emergence and persistence of resistant pathogens. While most existing research has focused on poultry, a significant knowledge gap remains regarding non-poultry meats such as beef and mutton. Previous antimicrobial surveillance efforts within Pakistan’s meat sector have predominantly focused on isolating single, targeted pathogens (such as *Salmonella* or *Staphylococcus aureus*) directly from raw meat or poultry samples [11,14]. Consequently, the complex ecological dynamics of multi-species biofilms residing on retail contact surfaces, and how these structural micro-environments drive metabolic vigor and multidrug resistance (MDR) in the non-poultry sector, remain a critical knowledge gap locally and globally [15,16]. Bridging this divide, this study serves as among the earliest integrated ecological analyses combining phylogenetic, metabolic, and resistance profiling of environmental bacteria recovered from Pakistan’s non-poultry meat-contact surfaces.

To address a significant knowledge gap in regional food safety, this study adopts a holistic approach to characterize the Biofilm-AMR nexus from a One Health perspective within retail meat environments in Wah Cantt, Pakistan. Specifically, we investigated the phylogenetic diversity, metabolic activity, and antimicrobial resistance profiles of bacterial isolates recovered from meat-contact surfaces, providing a comprehensive assessment of biofilm architecture and AMR dynamics.

## 3. Materials and Methods

### 3.1. Study Area and Design

This cross-sectional observational study was conducted in non-poultry meat retail outlets and butcher shops across different areas of Wah Cantt, Pakistan, to capture intra-city variation. To obtain a representative spatial snapshot of the retail landscape, a spatially stratified approach was designed to ensure that the recovered isolates reflect the broader ecological distribution of persistent pathogens within the city’s meat supply chain. The 30 retail shops surveyed were located in high-traffic areas typically experiencing moderate to high customer flow (10–30 or more customers per hour). Sanitation levels were generally moderate to poor, with hygiene practices neither strictly enforced nor entirely neglected. Cleaning routines were performed once to twice per day, and protective measures were limited: very few shopkeepers wore aprons, none wore gloves, and most handled currency with bare hands while also touching meat and surfaces. Ambient temperatures during the study period were consistently around 30 °C, creating conditions that were moderately hygienic but ecologically favorable for microbial persistence and biofilm formation.

This study was designed to explore the ecological population of biofilm-forming bacteria persisting on non-poultry meat-processing surfaces in Wah Cantt, providing a localized snapshot of their role as contamination reservoirs. By focusing on these persistent biofilm formers, we highlight their potential contribution to the dissemination of antimicrobial resistance within a One Health framework, providing an appropriate context for investigating biofilm-associated AMR.

### 3.2. Sample Size and Collection

Thirty random non-poultry (beef and mutton) butcher shops were selected to represent high-traffic and low-sanitation areas. A total of 300 isolates were initially recovered from 120 meat-contact surface swabs. From each shop, four environmental sites representing frequently contacted meat-processing surfaces were sampled: wooden cutting boards, knives, mincer machines, and weighing machine trays, using the sampling protocol. This multi-point sampling within each cluster was designed to capture the intra-shop variation in microbial biofilms while maintaining city-wide representativeness. The sample size of 120 swabs [17] was determined using the Cochran formula [18,19,20].

**n_0_ = (Z^2^ × p × (1 − p))/e^2^**

Assuming a 50% prevalence of resistant bacteria with a 9% margin of error and 95% confidence interval, the study will ensure it captures a representative snapshot of the retail environment. To focus on the most persistent threats within the One Health continuum, a phenotypic screening was conducted using the Tissue Culture Plate (TCP) assay. Only isolates exhibiting a strong biofilm-forming phenotype (*n* = 42) were selected for downstream molecular identification and comprehensive antimicrobial susceptibility testing. This targeted approach ensures that the study prioritizes ‘high-risk’ persistent strains that facilitate cross-contamination in food-processing environments.

### 3.3. Sampling Technique

Swab samples were collected from wooden chopping boards, knives, mincing machines, and weighing trays, all of which come into direct contact with meat. A standardized surface area of 10 cm^2^ at each site was swabbed with sterile cotton swabs moistened with 5 mL phosphate-buffered saline. Each sampled surface was considered a single sample unit.

Sampling was conducted under aseptic conditions, and swabs were immediately placed into sterile sample containers. All samples were transported to the microbiology laboratory under cold-chain conditions (4–8 °C) and processed within 1–2 h of collection.

### 3.4. Sample Processing, Isolation, and Purification of Bacteria

Swab extracts were serially diluted tenfold in sterile physiological saline and processed separately. Aliquots (1 mL) of each dilution (10^−2^ to 10^−6^) were inoculated into nutrient broth tubes and incubated overnight at 37 °C for enrichment. Subsequently, 0.1 mL aliquots from 10^−3^, 10^−4^, and 10^−5^ dilutions were spread onto nutrient agar plates and incubated aerobically at 37 °C for up to 48 h to recover aerobic mesophilic bacteria.

Following incubation, approximately 300 colonies were initially isolated from agar plates. Distinct colonies were purified by repeated streaking onto fresh nutrient agar to obtain pure cultures. Preliminary bacterial identification was based on colony morphology and Gram staining. Representative isolates were preserved as stock cultures in 40% (*v*/*v*) glycerol at −40 °C for further analysis.

### 3.5. Biofilm Formation Assays

**Figure 1:**
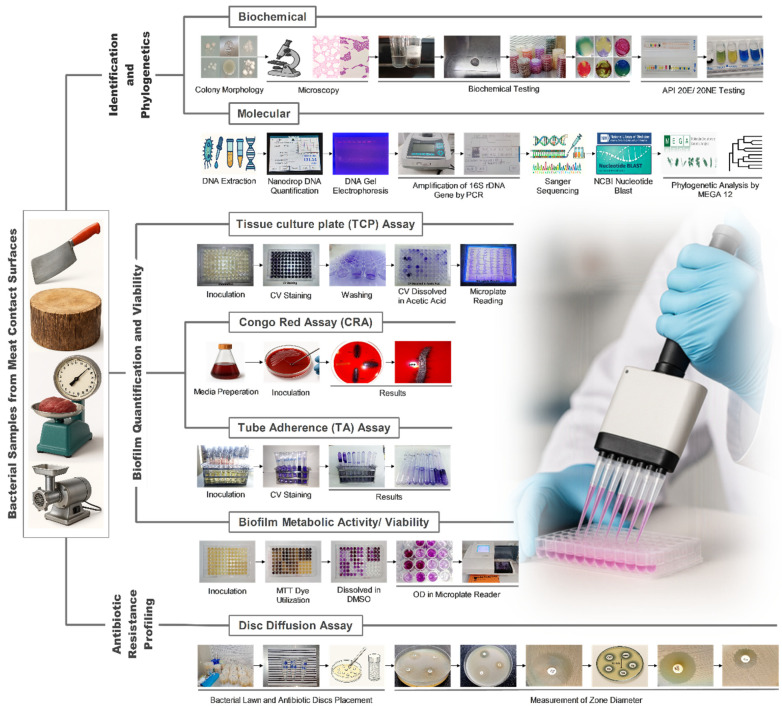
Research Snapshot.

Biofilm formation was assessed using established methodologies (Figure 1):

• Congo Red Agar Assay: Bacterial isolates were streaked onto Congo red agar plates composed of brain heart infusion agar supplemented with sucrose and Congo red dye. The inoculated plates were incubated at 37 °C for 24 h. Black colonies with a dry, crystalline appearance were interpreted as biofilm producers using the method described by Freeman et al. [21].
• Tube Adherence Assay: Bacterial isolates were inoculated into sterile test tubes containing tryptic soy broth and incubated at 37 °C for 24 h. After incubation, the tubes were gently washed three times with phosphate-buffered saline to remove non-adherent cells, then stained with 0.1% crystal violet. Biofilm formation was evaluated qualitatively based on the presence and intensity of a visible film lining the inner walls and bottom of the tubes, as previously described by Reddy et al. [22].
• Tissue Culture Plate Assay: Bacterial isolates were inoculated into sterile 96-well flat-bottom microtiter plates, with each well containing 200 µL of trypticase soy broth adjusted to the desired inoculum density (typically equivalent to a 0.5 McFarland standard). Plates were incubated statically at 37 °C for 24 h to allow biofilm formation. Following incubation, non-adherent cells were removed by washing the wells three times with 200 µL of phosphate-buffered saline. The plates were then air-dried and stained with 200 µL of 0.1% crystal violet for 15 min. Excess stain was gently rinsed off, and bound dye was subsequently solubilized with 200 µL of 30% acetic acid for 15 min. Biofilm biomass was quantified by measuring optical density at 570 nm with a microplate reader, using a cut-off OD (OD_C_), as described by Stepanović et al. [23].

$$\mathrm{Non-biofilm}\;\mathrm{producer}:\;\mathrm{OD}\;\leq \;{\mathrm{OD}}_{C}$$

$$\mathrm{Weak}\;\mathrm{biofilm}\;\mathrm{producer}:\;{\mathrm{OD}}_{C}\;\leq OD\;\leq \;2{\mathrm{OD}}_{C}$$

$$\mathrm{Moderate}\;\mathrm{biofilm}\;\mathrm{producer}:\;2{\mathrm{OD}}_{C}\;\leq OD\;\leq \;4{\mathrm{OD}}_{C}$$

$$\mathrm{Strong}\;\mathrm{biofilm}\;\mathrm{producer}:\;\mathrm{OD}>\;4{\mathrm{OD}}_{C}$$

### 3.6. Biochemical and Molecular Identification of Bacteria

Biofilm-forming purified isolates were biochemically identified through systematic biochemical characterization using standard microbiological procedures and API 20E/NE test strips (bioMérieux, Marcy-l’Étoile, France). The obtained data were interpreted according to the guidelines indicated in Bergey’s Manual of Determinative Bacteriology [24], as shown in Figure 1. A selected set of isolates was identified based on 16S rDNA gene sequencing due to their high biofilm-forming capacities. Genomic DNA extraction was performed using a modified Phenol-Chloroform/CTAB method [25] due to the robust biofilm-forming abilities of the isolates. The 16S rDNA gene was amplified using optimized PCR conditions by using universal primers 27F and 1492R [26]. PCR products were cleaned using GeneJET Gel Extraction Kit by Thermo Fisher Scientific^TM^, Vilnius, Lithuania. The cleaned PCR products were sent to 1st Base Laboratories in Malaysia for Sanger sequencing. The cleaned sequences were identified to the species level by using NCBI Blast (see Figure S1 in the Supplementary Data for additional visualization).

### 3.7. Phylogenetic Analysis

The ClustalW program was used to perform sequence alignment, and MEGA 12 was used to construct phylogenetic trees using the Neighbor-Joining method (1000 bootstrap replicates). All branches with bootstrap support <70% were collapsed to ensure the reliability of the taxonomic clades. Individual GenBank accession numbers for each isolate were integrated into the terminal node labels of the phylogenetic trees (Figure S4).

### 3.8. Antibiotic Susceptibility Testing

Kirby-Bauer disc diffusion method and minimum inhibitory concentration were used to perform antibiotic susceptibility testing, based on the recommendations of Clinical and Laboratory Standards Institute CLSI M100 (2020, 30th Edition). The antibiotics tested included Piperacillin (PRL), Ampicillin (AMP), Amoxicillin-Clavulanate (AUG), Piperacillin-Tazobactam (TPZ), Cefotaxime (CTX), Cefuroxime (CXM), Cefepime (CPM), Ceftazidime (CAZ), Cefazolin (CZ), Cefoxitin (FOX), Aztreonam (AM), Imipenem (IMP), Meropenem (MEM), Chloramphenicol (C.), Gentamicin (GM), Tobramycin (TN), Amikacin (AK), Ciprofloxacin (CIP), Levofloxacin (LEV), Norfloxacin (NOR), Ofloxacin (OFX), Colistin (COL), Trimethoprim-Sulfamethoxazole (SXT), Fosfomycin (FF), Tetracycline (TE), Nitrofurantoin (F), Vancomycin (VA), Rifampin (RA), Linezolid (LNZ), Teicoplanin (TEC), Erythromycin (E), and Clindamycin (DA).

### 3.9. Biofilm Biomass and Metabolic Activity Assessment

Biofilm formation was quantified using a combination of crystal violet staining for biomass [23] and an MTT reduction assay to assess metabolic activity. For metabolic activity assessment, the MTT assay [27] was performed with minor modifications. Each experiment was conducted in triplicate, and cell-free wells were included as controls to eliminate background interference.

### 3.10. Statistical Methods

Data analysis and visualization were conducted using Python (3.13) and R Studio. Biofilm production capacity was categorized based on established tissue culture plate thresholds. To rigorously validate the choice of inferential methods, data distributions were evaluated for normality using the Shapiro–Wilk test. The results confirmed that both OD CV and OD MTT datasets significantly deviated from a normal distribution (*p* < 0.05), thereby justifying the use of non-parametric frameworks. Consequently, Mann–Whitney U and Kruskal–Wallis H tests were employed to compare biofilm metrics across environmental sources and resistance profiles (MDR/XDR). The biomass-activity relationship was assessed using Spearman’s rank correlation. To mathematically decouple structural variables and evaluate predictors of multidrug resistance, an Ordinary Least Squares (OLS) multiple linear regression model was constructed to assess the impact of metabolic vigor and bulk biomass on the Multiple Antibiotic Resistance Index (MARI). Additionally, ecological community structuring was quantified using taxonomic Richness (S), the Shannon Diversity Index (H′), and Pielou’s Evenness (J′), while hierarchical clustering was applied to map the spatial signatures of antimicrobial resistance. Statistical significance for all tests was set at *p* < 0.05.

## 4. Results

### 4.1. Biofilm Formation and Resistance Correlation

The biofilm-forming potential of all 300 isolates was initially screened using the quantitative tissue culture plate method. Among these, 14% (*n* = 42) were identified as strong biofilm formers [12]. To further investigate the high-vigor phenotypes, a specialized subset of 44 isolates was selected, comprising 42 strong formers and 02 isolates identified as moderate and non-biofilm formers, serving as internal controls (LUB-219 and LUB-01) to provide comparative baselines. While limited, these controls offered biologically relevant anchors for our retail niche.

Further characterization of this high-risk subset (*n* = 42) using a tube assay and Congo red agar provided greater granularity of the biofilm matrix. Tube assay categorized 15.9% (*n* = 7) as strong and 40.9% (*n* = 18) as moderate formers. Meanwhile, Congo red agar method identified 47.7% (*n* = 21) as active slime formers, exhibiting the characteristic black-crystalline colony morphology (See Figure S2 in the Supplementary Data for additional visualization). This notable discordance between the qualitative visual methods (tube assay and Congo red agar) and the highly sensitive quantitative tissue culture plate (TCP) method highlights a known methodological limitation: qualitative assays are prone to high false-negative rates when evaluating robust environmental isolates that form microscopically thin, yet metabolically dense matrices.

**Figure 2:**
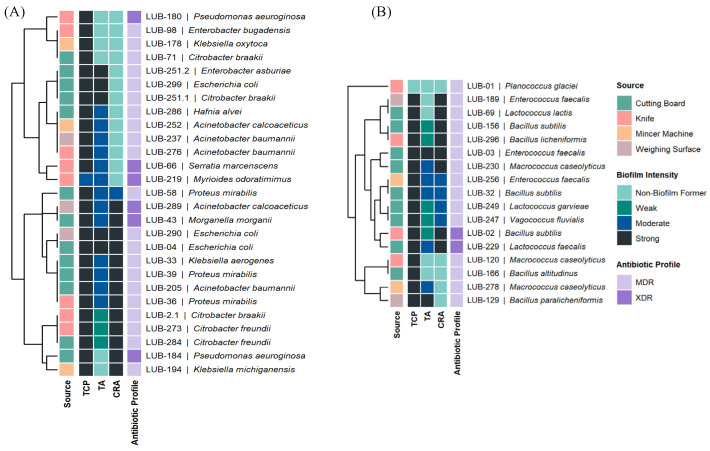
Integrated Phenotypic Clustering of Surface Isolates. (**A**) Gram-Negative Community: Dendrogram and heatmap illustrating relationships among ecological source, biofilm-forming intensity, and antimicrobial resistance (AMR) profiles. Hierarchical clustering identifies conserved resistance signatures in genera such as *Acinetobacter*, *Pseudomonas*, and *Escherichia*. (**B**) Gram-Positive Community: Clustering of *Macrococcus*, *Enterococcus*, and *Bacillus* species based on functional traits. Sidebars present categorical data from tissue culture plate, Tube assay, and Congo red agar assays, along with MDR and XDR classifications.

Overall, biofilm assays revealed that many isolates formed moderate to strong biofilms, particularly *Pseudomonas*, *Klebsiella*, *Acinetobacter*, and *Enterococcus*. Hierarchical clustering confirmed that strong biofilm-formers frequently overlapped with MDR/XDR phenotypes, suggesting a synergistic role of biofilm-mediated persistence and antimicrobial resistance (Figure 2).

### 4.2. Taxonomic Distribution and Source Characterization

As demonstrated in Table 1, porous materials like Wood (Cutting Boards) not only yielded the highest recovery rate of isolates (*n* = 97) but also fostered the greatest taxonomic richness among strong biofilm formers (S = 7, H′ = 1.53). This provides empirical evidence that surface porosity directly facilitates microbial establishment. Notably, Steel/Iron surfaces (Mincing Machines) exhibited a 100% MDR rate among their strong biofilm formers, paired with a high Evenness score (J′ = 0.96). This suggests that the mechanical stress of mincing devices selects for a highly resistant, uniformly balanced multi-family consortium rather than allowing a single species to dominate. While Enterobacteriaceae dominated most surfaces (comprising 50% of the biofilm formers on wood and steel), the Plastic/Mixed weighing surfaces showed an ecological shift, with Moraxellaceae becoming the predominant persisting family (40%).

A total of 44 bacterial isolates were analyzed, comprising 42 environmental test strains and two internal reference moderate-to-weak biofilm-forming benchmarks: LUB-219 (*Myrioides odoratimimus*) and LUB-01 (*Planococcus glaciei*). The isolates were recovered from critical meat-contact surfaces: Cutting Boards 47.7% (*n* = 21), Knives 27.3% (*n* = 12), Weighing Surfaces 13.6% (*n* = 6), and Mincer Machines 11.4% (*n* = 5). Based on the microscopic, biochemical, and molecular analysis, 17 diverse genera were reported, and these include: *Enterococcus* spp., *Escherichia* spp., *Bacillus* spp., *Acinetobacter* spp., *Klebsiella* spp., *Pseudomonas* spp., *Enterobacter* spp., *Citrobacter* spp., *Lactococcus* spp., *Macrococcus* spp., *Vagococcus* spp., *Planococcus* spp., *Hafnia* spp., *Myroides* spp., *Morganella* spp., *Serratia* spp., and *Proteus* spp. belonging to approximately 10 different families. Taxonomical mapping identified *Enterobacteriaceae* as the dominant family (*n* = 19), particularly on Cutting Boards and Weighing Surfaces, while *Moraxellaceae* and *Staphylococcaceae* were prevalent on Knives and Mincer Machines.

Enterobacteriaceae dominate, accounting for approximately 43% of all isolates (primarily *Citrobacter*, *Escherichia*, *Klebsiella*, and *Proteus*). *Moraxellaceae*, *Bacillaceae*, and *Enterococcaceae* each contribute 11.4%; Streptococcaceae and Staphylococcaceae are available at 6.8%; Pseudomonadaceae is 4.5%; while Planococcaceae and Flavobacteriaceae are the least represented (2.3% each), as shown in Figure S3 in the Supplementary Data. The overall percentages of Gram-negative and Gram-positive bacteria among total bacterial isolates were 61.4% and 38.6%, respectively.

• ^a^ Richness (S): Total number of distinct bacterial families identified among the strong biofilm-producing isolates per surface.
• ^b^ Shannon-Wiener Index (H′): Calculated as $H^{\prime }=-\sum p_{i}lnp_{i}$, representing taxonomic diversity within the biofilm-forming consortium.
• ^c^ Pielou’s Evenness (J′): Calculated as $J^{\prime }=H^{\prime }lnS$. High evenness values (>0.75) indicate that resistance traits are not confined to a single family but are distributed across a balanced multi-family community.

### 4.3. Phylogenetic Relationships

Phylogenetic analysis of 27 Gram-negative isolates (Figure S4A) revealed six well-supported clades, with tight clustering potentially reflecting shared ecological niches and survival strategies. *Proteus mirabilis* (100% bootstrap support) and the *Acinetobacter baumannii*/*calcoaceticus* complex (96–98%) form highly compact groups, which we hypothesize is consistent with conserved exopolysaccharide synthesis. This clustering underscores the risk of specialized antibiotic-resistant lineages persisting on retail meat-contact surfaces. *Citrobacter* spp. and *Pseudomonas aeruginosa* occupy distinct branches, suggesting lineage-specific adaptations that enhance persistence under sanitation stresses. The *Escherichia coli* and mixed *Enterobacteriaceae* clades highlight potential horizontal gene exchange, which could facilitate the dissemination of resistance loci, though this requires whole-genome validation.

In the Gram-positive phylogenetic tree (Figure S4B), *Bacillus* spp. cluster tightly (85–90 bootstrap), indicating robust evolutionary relationships that mirror their shared biofilm-forming capacities. Similarly, *Macrococcus*, *Planococcus*, and *Enterococcus faecalis* form cohesive branches indicative of distinct environmental-persistence phenotypes. Together, these relationships demonstrate that evolutionary proximity correlates with common mechanisms for surface attachment and the maintenance of resistance determinants. The triad of phylogenetic adaptation, biofilm-forming capacity, and antibiotic resistance creates a perfect storm for persistent contamination, clearly illustrating the limitations of surface sanitation. However, we emphasize that 16S rDNA data primarily reflect taxonomic relatedness; advanced comparative genomics (e.g., Whole Genome Sequencing) is strictly essential in future studies to definitively prove specific horizontal gene transfer events and the sharing of specific resistance loci.

### 4.4. Antibiotic Resistance Polarity and Surface Hotspots

**Figure 3:**
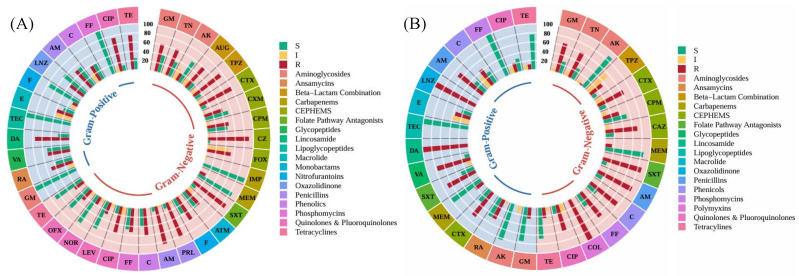
Antibiotic Response Polarity Across 44 Environmental Isolates. (**A**) Polarity plot derived from disk diffusion assay showing % resistance by antibiotic class. (**B**) Polarity plot for minimum inhibitory concentration values, categorizing bacteria as sensitive (S), intermediate (I), and resistant (R).

Antimicrobial profiling via disk diffusion and minimum inhibitory concentration revealed a high burden of antimicrobial resistance. Resistance was most prevalent against Penicillins (Ampicillin: 84.2% in Gram-negatives, 50% in Gram-positives) and Cephems (Cefazolin: 89.5%; Cefotaxime: 79.2%). Carbapenems remained largely effective, with 0% resistance to Imipenem and only 14.8% to Meropenem (Figure 3).

### 4.5. Clustering of Resistotypes and Critical Spatial AMR Signatures

**Figure 4:**
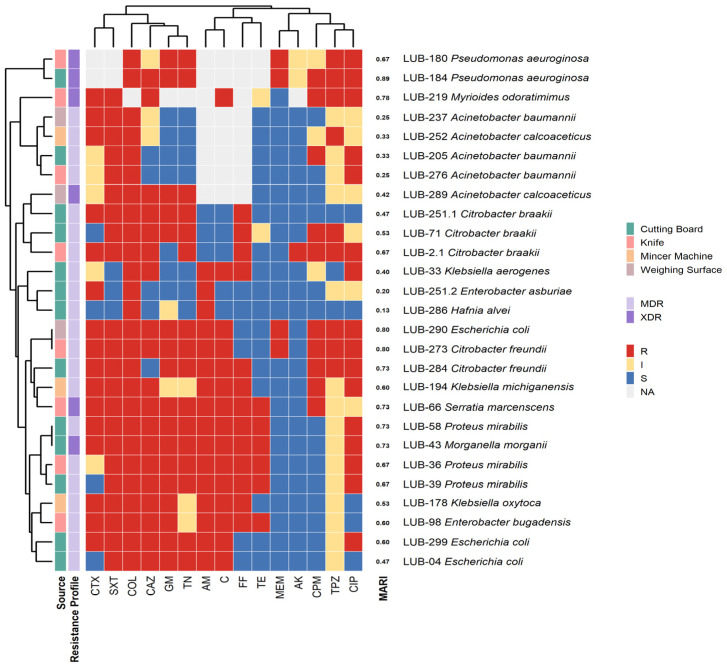
Resistance-Susceptibility Heatmap of Gram-Negative Meat Isolates: Integrated analysis of phenotypic clustering and antibiogram data for Gram-negative genera recovered from non-poultry meat-contact surfaces. The horizontal axis displays the minimum inhibitory concentrations of 15 antimicrobial agents, while the vertical axis represents hierarchical clustering of isolates based on Euclidean distance. The color gradient illustrates the transition from susceptible (cool colors) to XDR (warm colors) phenotypes. This Heatmap traces susceptibility trends and identifies rare resistance signals, such as colistin resistance, within the informal meat supply chain.

**Figure 5:**
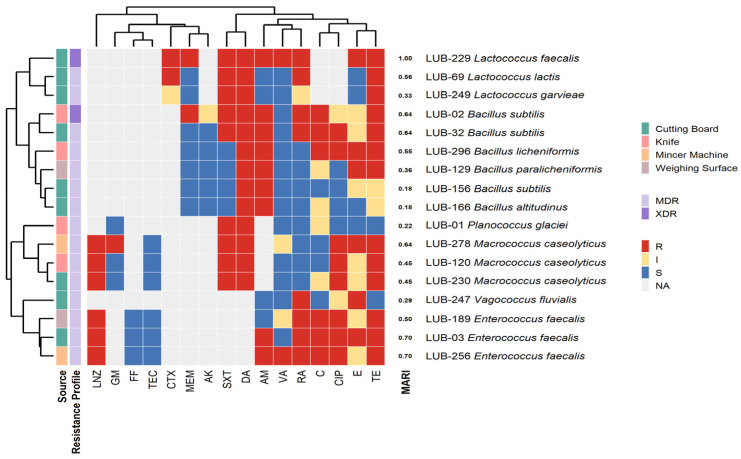
Resistance-Susceptibility Phenogram of Gram-Positive Meat Isolates. The branching patterns of the phenogram illustrate the functional relatedness of isolates based on their multidrug-resistance (MDR) signatures. Integration with minimum inhibitory concentration data highlights critical clusters of resistance to last-resort antibiotics, including Linezolid.

Heatmap clustering demonstrated distinct resistance phenotypes. Gram-negative isolates (*Pseudomonas aeruginosa*, *Acinetobacter baumannii*, *Klebsiella* spp.) exhibited extensive resistance to last-line agents, with MARI values approaching 1.00, indicating high antibiotic pressure. Gram-positive isolates (*Enterococcus faecalis*, *Macrococcus caseolyticus*) also showed elevated MARI values (0.70–1.00), while some *Bacillus* and *Planococcus* isolates displayed lower indices (0.16–0.38). According to the international consensus definitions, MDR is defined as acquired non-susceptibility to at least one agent in three or more antimicrobial categories. XDR represents a more severe resistance profile, defined as non-susceptibility to at least one agent in all but two or fewer antimicrobial categories [28]. Hierarchical clustering of resistance profiles and MAR indices revealed a high-risk spatial signature, with 81.8% (*n* = 36) of isolates exceeding the 0.2 MAR threshold and being confirmed as MDR, while 18.2% (*n* = 8) reached XDR status. The Multiple Antibiotic Resistance Index (MARI) averaged 0.49 ± 0.19, with XDR isolates demonstrating the greatest breadth of resistance, predominantly recovered from cutting boards and knives (Figure 4 and Figure 5). The high prevalence of MDR and XDR phenotypes among our strong biofilm-forming isolates is consistent with recent findings in Peshawar and Lahore, where multidrug resistance was strongly correlated with robust biofilm production in beef and mutton supply chains [11,29]. Cutting Board isolates exhibited the widest resistance spectra, spanning 10 antibiotic classes (Table 1). When compared to minimum inhibitory concentration-based heatmaps, disc diffusion data showed strong concordance, with both methods identifying broad-spectrum resistance and clustering of MDR/XDR phenotypes. MIC analysis provided quantitative confirmation of resistance intensity, while disc diffusion highlighted categorical resistance patterns. Together, these approaches reinforced the finding that resistant isolates were not only diverse but also consistently distributed across Gram-negative and Gram-positive groups, with biofilm-forming strains frequently overlapping with MDR/XDR profiles (See Figures S5 and S6 in Supplementary Data). Mapping the observed critical resistance phenotypes against the WHO AWaRe framework (Table 2) revealed a severe public health threat, as 79.5% of these phenotypes compromised global ‘Reserve’ category antibiotics like colistin and Linezolid [30]. The remaining phenotypes predominantly threatened the ‘Watch’ category. Crucially, ecological stratification demonstrated that mechanical mincers exclusively harbored 100% ‘Reserve’-threat phenotypes, indicating that the most globally prioritized resistance traits are heavily concentrated within specific processing machinery [31].

### 4.6. Quantitative Biofilm Dynamics: Biomass vs. Metabolic Activity

**Figure 6:**
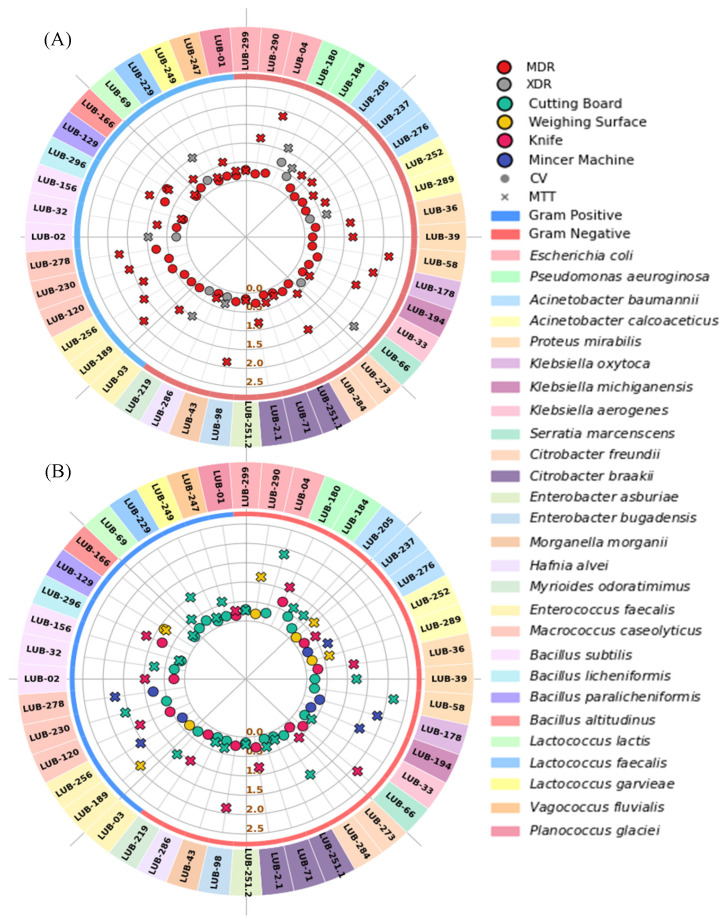
Quantitative Characterization of Biofilm Biomass and Metabolic Activity. (**A**) Scatter plot showing the strong positive correlation (ρ = 0.656, *p* < 0.001) between total biomass (OD_CV_) and metabolic activity (OD_MTT_) for all 44 isolates. (**B**) Comparison of metabolic activity (OD_MTT_) across the four contact surfaces, identifying Mincer Machines as significantly high-activity zones (*p* < 0.05).

Quantitative analysis of the 42 strong producers revealed a mean biomass (OD_CV_) of 0.298 ± 0.18 and a mean metabolic activity (OD_MTT_) of 0.967 ± 0.59. These values were significantly higher than those of the control group (Mean OD_CV_: 0.178; OD_MTT_: 0.641). A strong positive correlation was observed between biomass and metabolic activity (Spearman’s ρ = 0.656, *p* < 0.001), confirming that the structural matrix supports high cellular metabolism (Figure 6). Mincer Machine isolates showed the highest mean metabolic activity (1.492 ± 0.45), significantly higher than on other surfaces (Kruskal–Wallis, *p* = 0.035). Knives showed moderate metabolic activity (1.05 ± 0.62), while biomass levels were significantly lower than those in the internal components of mincers, suggesting that mincers’ complex architecture supports more mature, metabolically active microbial communities.

### 4.7. Integrated Analysis of Biofilm Phenotypes, Metabolic Fitness, and Antimicrobial Resistance Associations

**Figure 7:**
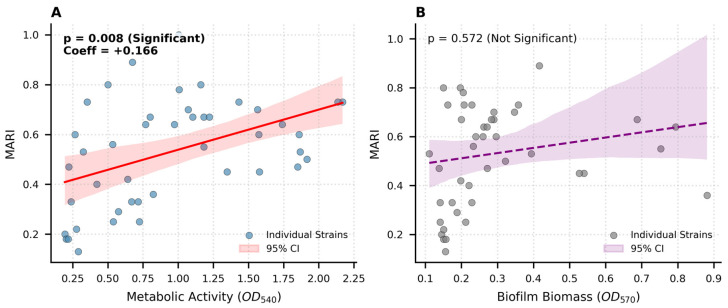
Ordinary Least Squares (OLS) regression analysis predicting antimicrobial resistance from biofilm physiological metrics. (**A**) Scatter plot illustrating a statistically significant positive relationship (*p* = 0.008) between metabolic viability (MTT OD_540_) and the Multiple Antibiotic Resistance Index (MARI). The solid red regression line demonstrates that highly viable, actively respiring cellular populations are strong predictors of elevated multidrug resistance. (**B**) Scatter plot evaluating total structural biomass (CV OD_570_) against MARI. A dashed regression line has been applied to find out the overall correlation, revealing a flat, non-significant trend (*p* = 0.572). This perfectly illustrates the inherent assay discordance when compared to Panel 7A; the sheer volume of the extracellular matrix does not reliably dictate an isolate’s antimicrobial resistance profile.

In this study, the relationship between physiological traits and antibiotic resistance profiles was evaluated using the MARI. To evaluate the factors driving multidrug resistance, an Ordinary Least Squares (OLS) regression analysis assessed the MARI against structural biomass (CV OD_570_), metabolic activity (MTT OD_540_), surface types, and Gram status. The overall model was statistically significant (*p* = 0.022, R^2^ = 0.258). A statistically significant positive correlation (Figure 7A) was observed between metabolic activity (measured via MTT assay) and the MARI (*r* = +0.166, *p* = 0.008), suggesting that higher metabolic rates may support the energetic demands of multidrug resistance mechanisms, such as the expression of efflux pumps or enzymatic inactivation. Conversely, no significant correlation (Figure 7B) was found between biofilm biomass (measured via Crystal Violet staining) and the MARI (*p* = 0.572). These findings indicate that while metabolic vigor is closely linked to the breadth of resistance of the isolates, the quantitative production of biofilm matrix does not reliably predict the degree of multidrug resistance in the tested strains. This suggests that resistance in these isolates may be governed more by specific genetic determinants or metabolic adaptations rather than physical sequestration within a robust biofilm matrix.

**Figure 8:**
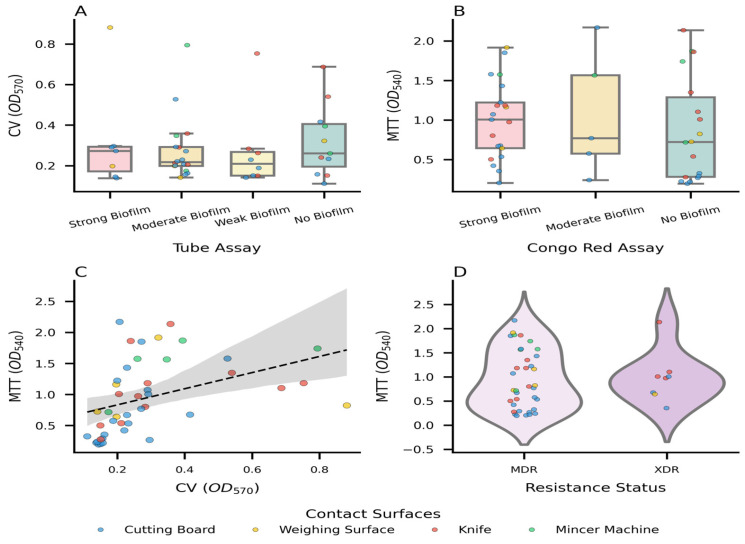
Biofilm dynamics, metabolic fitness, and antimicrobial resistance across the combined dataset. Panels (**A**,**B**) illustrate assay discordance, confirming that extracellular matrix abundance does not universally predict metabolic activity. Panel (**C**) demonstrates a positive correlation between biomass and viability, while Panel (**D**) shows that MDR/XDR phenotypes maintain elevated metabolic fitness, underscoring their ecological persistence on food-contact surfaces. (**A**) Boxplots with overlaid strip plots display total structural biomass (CV OD_570_) across Tube Assay grades, color-coded by surface type. (**B**) Boxplots and strip plots show metabolic viability (MTT OD_540_) across Congo Red Assay classifications, highlighting the inherent assay discordance when compared to Panel (**A**). (**C**) A scatter plot evaluates structural biomass versus metabolic viability; a dashed regression line has been applied to find out the overall correlation, demonstrating a positive linear trend despite individual discordance. (**D**) Violin plots with strip plots depict the density distribution of metabolic fitness stratified by Multidrug-Resistant (MDR) and Extensively Drug-Resistant (XDR) phenotypes.

Complementing these regression analyses, Figure 8 provides a broader synthesis of biofilm phenotypes and metabolic fitness across the complete dataset, highlighting assay discordance, trait correlations, and the influence of resistance profiles on physiological vigor. Figure 8 synthesizes the collective biofilm phenotypes and metabolic fitness of Gram-negative and Gram-positive isolates recovered from diverse meat-contact surfaces. Panels A and B highlight the inherent phenomenon of assay discordance, with boxplots and overlaid strip plots demonstrating the distribution of structural biomass (CV OD_570_) across Tube Assay grades (Figure 8A) and metabolic viability (MTT OD_540_) across Congo Red Assay categories (Figure 8B). The divergence in data spread across these panels confirms that robust extracellular matrix production does not universally correlate with elevated respiratory activity, underscoring the need for complementary assays to obtain a complete physiological profile. To evaluate the macroscopic relationship between these traits, a scatter plot with regression analysis (Figure 8C) revealed a positive, linear correlation, indicating that isolates with larger biofilm matrices generally harbor proportionally more metabolically active cells. Finally, violin plots overlaid with strip plots (Figure 8D) summarized metabolic fitness in relation to antimicrobial resistance status, demonstrating that both MDR and XDR isolates sustain elevated respiratory activity, with XDR strains exhibiting broader variability. Collectively, these findings establish that biofilm matrix production and metabolic vigor are interdependent yet distinct traits and that resistance phenotypes further amplify the ecological persistence of foodborne isolates.

## 5. Discussion

Under the One Health framework, addressing AMR requires granular data from diverse environments. This study provides localized evidence underscoring the need for expanded surveillance in the non-poultry sector—a niche that remains critically underexplored compared to industrial poultry chains. The widespread colonization of meat-contact surfaces by strong biofilm-forming, MDR bacteria poses a significant challenge to food safety, acting as a resilient reservoir for cross-contamination and zoonotic transmission within localized retail meat markets. Furthermore, the methodological discordance observed, in which qualitative visual assays significantly underestimated biofilm capacity compared to the quantitative TCP assay, demonstrates that relying on subjective EPS detection fundamentally misclassifies these hyper-viable reservoirs [23,33,34]. This finding emphasizes the necessity of quantitative metabolic screening to avoid the under-reporting of high-risk microbial clades.

### 5.1. Phylogenetic Resolution and Taxonomic Diversity Analysis

Phylogenetic clustering indicates that surface-adapted isolates may share conserved phenotypic determinants for resilience. In the comparative analysis of bacterial groups (Figure S4), Gram-negative isolates exhibited greater species richness (H′ = 2.68), suggesting a taxonomically complex “resistome” where multiple species coexist. This high diversity may facilitate an environment conducive to horizontal gene transfer (HGT) of MDR/XDR traits. However, while we observed lower bootstrap support (avg. 74%) for some Gram-negative clusters, Gram-positive taxa showed more robust phylogenetic resolution (avg. 82%), suggesting stable environmental adaptation within these clades. We acknowledge that 16S rDNA sequencing is insufficient to definitively confirm evolutionary mechanisms; consequently, we have framed our observations of “ecological clustering” as phenotypic adaptations rather than confirmed genomic convergence. The high prevalence (43%) of enteric bacteria such as Escherichia, Klebsiella, Proteus, and Serratia [17] points to significant fecal contamination and a breakdown in hygienic abattoir-to-retail transitions—a core One Health concern.

### 5.2. The Biofilm-Resistance Nexus

Recent reviews emphasized that MDR and XDR isolates often evolve through efflux pump overexpression, target modification, and adaptive resistance mechanisms, all of which are potentiated within biofilm matrices [35,36]. The strong correlation between total biomass (OD_CV_) and metabolic activity (OD_MTT_) indicates that these biofilms are not merely physical barriers but active biological reservoirs. The concentration of XDR strains on Cutting Boards and Knives suggests that the biofilm matrix facilitates a “protective niche” where bacteria can maintain high metabolic vigor even under chemical stress [16,37,38]. These biofilms likely serve as sites for horizontal gene transfer (HGT), where mobile resistance elements can move between genera (e.g., from *Escherichia* to *Acinetobacter*). It poses a direct risk of introducing resistant pathogens into the food supply. While biomass did not differ significantly between MDR and XDR groups, the baseline of “strong” biofilm formation across all 42 test strains (Figure 2) indicates that biofilm-mediated tolerance is a universal strategy for MDR survival in these niches.

The finding that mincer machine isolates exhibit significantly higher metabolic activity despite having biomass similar to that of other surfaces suggests a specialized metabolic adaptation. The complex internal geometry and organic (meat) residue accumulation in these machines provide a nutrient-rich environment that sustains highly active biofilms and prevents effective surface sterilization. These “hotspots” are likely the primary sources of persistent contamination in the local meat processing chain. Together, these findings highlight that the biofilm–resistance nexus is a critical driver of antimicrobial resistance persistence in non-poultry meat environments. Crucially, our regression data provide deeper ecological insight, revealing that the distinct mechanical shear stresses and microtopographies of these environments actively shape the resistome by selecting for extreme, localized metabolic fitness rather than uniform physical barriers [39].

### 5.3. Public Health Implications of XDR Reservoirs

The detection of resistance to last-resort antibiotics, such as Polymyxins (Colistin) and Oxazolidinones (Linezolid), on common surfaces like weighing scales and knives is highly concerning. The high MARI scores (avg. 0.49) confirm that the facility environment is under constant selective pressure, favoring the persistence of highly resistant phenotypes, and is consistent with global reports of biofilm-mediated escalation of resistance. The detection of XDR and the high prevalence of MDR pathogens in a food-related environment raise alarm for both food safety regulators and public health authorities. The observed resistance trends among biofilm-forming isolates underscore the complex interplay between microbial ecology, surface colonization, and antimicrobial pressure. Biofilms are increasingly recognized as reservoirs for MDR and XDR phenotypes, with their structural and metabolic adaptations contributing to therapeutic failure in clinical and food-contact environments [40].

Variations in biofilm formation across meat-contact surfaces highlight the role of micro-environmental factors in pathogen persistence. Organic-rich surfaces, such as knives and cutting boards, serve as hotspots for biofilm-embedded MDR and XDR pathogens, whereas non-organic surfaces show lower activity due to reduced moisture and nutrient retention. The spatial distribution of resistant isolates in our study, particularly the concentration of XDR isolates on knives and cutting boards, aligns with the reported data [41], which emphasized the environmental persistence of MDR/XDR bacteria in water and surface samples, often linked to inadequate sanitation and biofilm resilience. The superior biofilm metrics of XDR isolates suggest evolutionary selection for enhanced protection, reinforcing the need for targeted sanitation that addresses both material properties and microbial dynamics. To promote global antibiotic stewardship, the isolates in this study were categorized using the WHO AWaRe framework, a critical tool for monitoring and discouraging inappropriate antibiotic use [42]. The finding that 79.5% of critical phenotypes, escalating to an absolute 100% on mechanical mincers, compromise WHO ‘Reserve’ antibiotics highlights a severe biosecurity vulnerability in retail meat processing [43]. These dynamic micro-environments likely exert extreme selective pressures through mechanical stress and complex micro-topographies, fostering metabolically vigorous biofilms that retain costly resistance elements against last-resort therapeutics [31]. From a One Health perspective, the continuous exposure of raw meat to these ‘Reserve-threat’ biofilms creates a high-risk transmission vector into the community food chain [44], exposing critical failures in traditional sanitation protocols and necessitating urgent, ecology-based interventions in line with international biosecurity frameworks [7]. Specifically, future interventions must test targeted anti-biofilm agents—such as matrix-degrading enzymes (e.g., DNases) and lytic bacteriophages—to dismantle these resilient communities where traditional biocides fail [27,45]. Future research should integrate metagenomics and surface metrology to further elucidate these ecological interactions.

### 5.4. Methodological and Genomic Limitations

While this study identifies critical local phenotypic trends regarding the biofilm-resistance nexus, specific genomic and methodological limitations must be acknowledged. First, the molecular analysis was confined to 16S rDNA sequencing, which lacks the high resolution required to confirm convergent evolutionary adaptation definitively. Second, while our phenotypic assays flagged severe resistance to last-resort antibiotics (e.g., colistin and Linezolid), this study lacked gene-level validation (e.g., screening for *mcr-1* or *cfr* genes). Finally, the absence of negative controls (e.g., blank samples) during initial PCR amplification represents a methodological constraint that should be rigorously standardized in future surveillance. Future validation utilizing Whole Genome Sequencing (WGS), Average Nucleotide Identity (ANI), or Multi-Locus Sequence Typing (MLST) is strictly necessary to substantiate these evolutionary claims [46].

## 6. Conclusions

This study establishes that retail meat-contact surfaces—specifically mechanical mincers and porous cutting boards—function as active ecological reservoirs for extensively drug-resistant (XDR) pathogens. Our findings provide a critical mechanistic shift in the Biofilm-AMR nexus: while biofilm biomass and cellular activity are strongly correlated (*p* < 0.001), Ordinary Least Squares (OLS) regression identifies metabolic vigor as the primary independent predictor of antimicrobial resistance severity (*p* = 0.008). This demonstrates that the “quality” of cellular respiration, rather than the physical “quantity” of the biofilm matrix, dictates the survival threshold of pathogens under antibiotic pressure.

The alarming 100% compromise of WHO Reserve category antibiotics (including Colistin and Linezolid) on mechanical processing equipment highlights a systemic failure in current sanitation protocols, which prioritize visual cleanliness over microbiological safety. While phylogenetic evidence identifies shared environmental niches among *Enterobacteriaceae* and *Acinetobacter* clades, the detection of last-resort resistance phenotypes necessitates urgent genomic surveillance to map the horizontal transfer of *mcr* and *cfr* determinants. Ultimately, these results necessitate a transition toward ecology-based biosecurity interventions, such as matrix-degrading enzymes and lytic bacteriophages, to disrupt the persistent transmission networks within the global food supply chain.

## Figures and Tables

**Table 1:** Surface-specific distribution, ecological indices, and antimicrobial resistance profiles of recovered environmental consortia. Data highlights the relationship between physical retail surfaces (microenvironments) and the establishment of highly resistant biofilm communities. Ecological structuring is defined by ^a^ Richness (S), ^b^ Shannon diversity (H′), and ^c^ Evenness (J′). Abbreviations: N, total swabs; n, total isolates; MDR, Multidrug-Resistant; XDR, Extensively Drug-Resistant; MAR, Multiple Antibiotic Resistance.

Surface Material	Swabs (N)	Total Recovered Isolates (n)	Strong Biofilm Formers	MDR (%)	XDR (%)	Mean MAR Index	Richness (S) ^a^	Shannon (H′) ^b^	Evenness (J′) ^c^	Predominant Family (%)
Wood (Cutting Boards)	30	97	22	86%	14%	0.51	7	1.53	0.79	*Enterobacteriaceae* (50.0%)
Stainless Steel (Knives)	30	96	10	70%	30%	0.6	5	1.36	0.84	*Enterobacteriaceae* (50.0%)
Steel/Iron (Mincing Machines)	30	34	5	100%	0%	0.56	4	1.33	0.96	*Enterobacteriaceae* (40.0%)
Plastic/Mixed (Weighing Surface)	30	73	5	80%	20%	0.45	4	1.33	0.96	*Moraxellaceae* (40.0%)
Total/Overall	120	300	42	-	-	-	-	-	-	-

**Table 2:** Integrated taxonomic, metabolic, and antimicrobial resistance profiles of high-risk biofilm formers across retail meat-contact surfaces. The data illustrate the distribution of bacterial families across surface niches and show that quantitative biofilm metrics (mean physical biomass and metabolic viability) correlate with the severity of multidrug resistance. Critical resistance phenotypes are classified by their highest compromised threat level according to the World Health Organization (WHO) AWaRe framework [30], highlighting the distribution of isolates that threaten global “Watch” and “Reserve” therapeutics.

Surface Niche	Bacterial Family	Strong Biofilm Formers (*n* = 42)	Resistance Classes (n)	Critical Resistance Phenotype	Highest AWaRe Category ^a^ Compromised.	Mean MAR Index	Mean Biomass (OD_570_ ± SD)	Mean Viability (OD_540_ ± SD)
**Wood** **(Cutting Boards)**	*Enterobacteriaceae*	11	10	Polymyxins, Cephalosporins, Fluoroquinolones	Reserve	0.51	0.19 ± 0.06	0.79 ± 0.73
	*Bacillaceae*	3	7	Fluoroquinolones	Watch	0.33	0.19 ± 0.07	0.40 ± 0.32
	*Streptococcaceae*	3	9	Carbapenems, Cephalosporins, Glycopeptides	Watch	0.63	0.22 ± 0.08	0.59 ± 0.39
	*Enterococcaceae*	2	7	Fluoroquinolones, Linezolid (Oxazolidinones), Macrolides	Reserve	0.49	0.24 ± 0.07	0.82 ± 0.35
	*Moraxellaceae*	1	4	Polymyxins, Cephalosporins, Fluoroquinolones	Reserve	0.33	0.23	0.67
	*Pseudomonadaceae*	1	6	Carbapenems, Polymyxins, Cephalosporins	Reserve	0.89	0.42	0.68
	*Staphylococcaceae*	1	5	Fluoroquinolones, Linezolid (Oxazolidinones)	Reserve	0.45	0.53	1.58
**Stainless Steel ** **(Knives)**	*Enterobacteriaceae*	5	10	Carbapenems, Polymyxins, Cephalosporins	Reserve	0.69	0.26 ± 0.08	1.30 ± 0.69
	*Bacillaceae*	2	9	Carbapenems, Fluoroquinolones, Macrolides	Watch	0.59	0.51 ± 0.35	1.08 ± 0.15
	*Moraxellaceae*	1	3	Polymyxins, Fluoroquinolones	Reserve	0.25	0.21	0.54
	*Pseudomonadaceae*	1	5	Carbapenems, Polymyxins, Fluoroquinolones	Reserve	0.67	0.69	1.1
	*Staphylococcaceae*	1	5	Fluoroquinolones, Linezolid (Oxazolidinones)	Reserve	0.45	0.54	1.35
**Steel/Iron ** **(Mincing Machines)**	*Enterobacteriaceae*	2	8	Polymyxins, Cephalosporins, Fluoroquinolones	Reserve	0.56	0.33 ± 0.09	1.72 ± 0.21
	*Enterococcaceae*	1	7	Polymyxins, Fluoroquinolones, Glycopeptides	Reserve	0.7	0.35	1.56
	*Moraxellaceae*	1	4	Polymyxins, Cephalosporins, Beta-Lactam/Inhibitors	Reserve	0.33	0.17	0.72
	*Staphylococcaceae*	1	7	Fluoroquinolones, Glycopeptides, Linezolid (Oxazolidinones)	Reserve	0.64	0.79	1.74
**Plastic/Mixed ** **(Weighing Surface)**	*Moraxellaceae*	2	4	Polymyxins, Cephalosporins, Aminoglycosides	Reserve	0.33	0.17 ± 0.04	0.68 ± 0.06
	*Bacillaceae*	1	4	Macrolides	Watch	0.36	0.88	0.82
	*Enterobacteriaceae*	1	9	Carbapenems, Polymyxins, Cephalosporins	Reserve	0.8	0.2	1.16
	*Enterococcaceae*	1	5	Fluoroquinolones, Linezolid (Oxazolidinones)	Reserve	0.5	0.32	1.92

^a^ AWaRe Category: Antibiotics categorized according to the WHO 2023 AWaRe (Access, Watch, Reserve) classification [32]. Access: First-line or second-line choices for common infections; Watch: Highest priority agents with greater resistance potential; Reserve: “Last-resort” antibiotics reserved for MDR/XDR infections.

## Data Availability

The original contributions presented in this study are included in the article/Supplementary Material. Further inquiries can be directed to the corresponding author.

## Supplementary Materials

The following supporting information can be downloaded at: https://www.mdpi.com/article/10.3390/microorganisms14051051/s1, Figure S1. PCR amplification of 16S rDNA gene sequences from selected isolates. Figure S2. Distribution of biofilm-forming potential among isolates as determined by different phenotypic assays. Figure S3: Taxonomic distribution and diversity indices of the isolated microbial community. Figure S4: Molecular phylogenetic characterization of the bacterial community isolated from meat-contact surfaces. Figure S5: Spatial heat grid analysis of Gram-negative isolates based on antimicrobial susceptibility profiling using the Disc Diffusion method. Figure S6: Antibiogram heatmap and hierarchical clustering of Gram-positive isolates using disc diffusion assay.
