# Supplementary material for: Persistence and Risk Assessment of Biofilm-Forming MDR and XDR Bacteria on Non-Poultry Meat Contact Surfaces in Wah Cantt, Pakistan
Source: Microorganisms. 2026 May 7;14(5):1051. doi: 10.3390/microorganisms14051051 (PMC13209423; doi:10.3390/microorganisms14051051)
Supplement: Supplementary file 1 [file microorganisms-14-01051-s001.zip › microorganisms-4274859-supplementary.pdf]

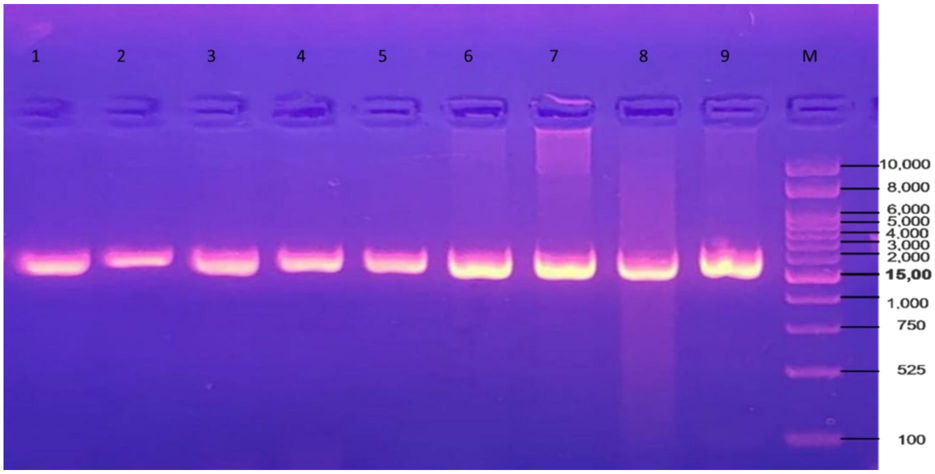

Figure S1. PCR amplification of 16S rDNA gene sequences from selected isolates. From left to right (lanes 1–10), meat contact surface isolates are: (90 LUB (*Escherichia coli*), 299 LUB (*Escherichia coli*), 43 LUB (*Morganella morganii*), 39 LUB (*Proteus mirabilis*), 2.1 LUB (*Citrobacter braakii*), 180 LUB (*Pseudomonas aeruginosa*), 184 LUB (*Pseudomonas aeruginosa*), 219 LUB (*Myroides odoratimimus*), 129 LUB (*Bacillus paralicheniformis*), and a DNA ladder.

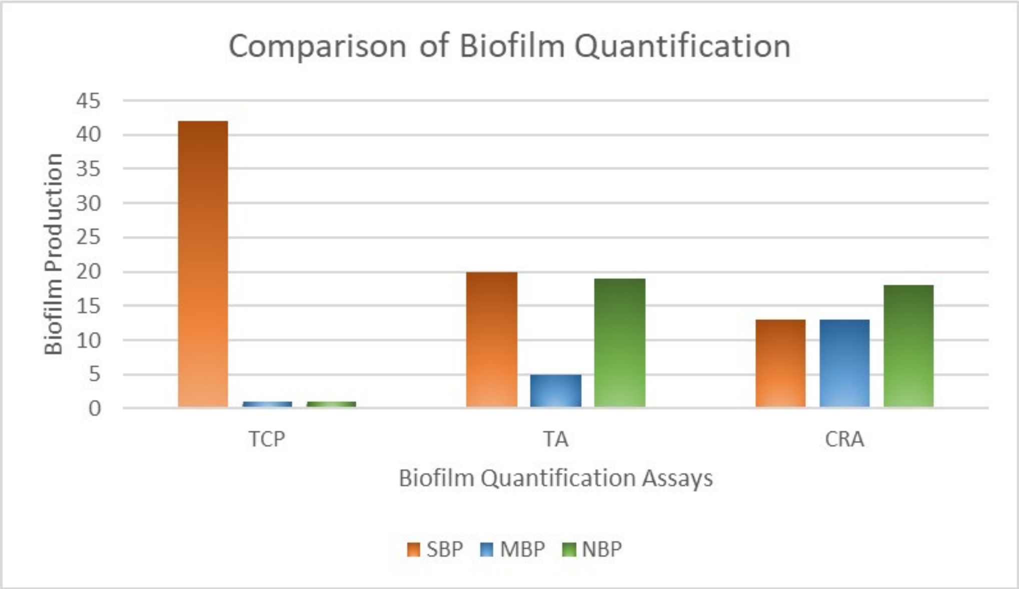

Figure S2. **Distribution of biofilm-forming potential among isolates as determined by different phenotypic assays.** The bar chart illustrates the categorical classification of biofilm production across the total microbial population (including both Gram-positive and Gram-negative isolates) using three screening methods: Tissue Culture Plate (TCP), Tube Adherence (TA), and Congo Red Agar (CRA). Isolates were stratified into SBP (Strong Biofilm Producers), MBP (Moderate Biofilm Producers), and NBP (Non/Weak Biofilm Producers) based on established optical density and phenotypic markers. The data demonstrate a higher detection frequency of the strong biofilm phenotype using the TCP method (n=42) than with the qualitative TA (n=20) and CRA (n=13) assays.

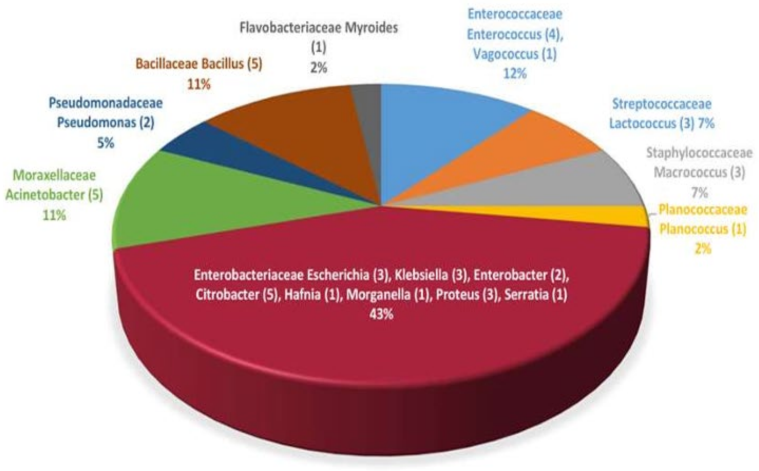

Group	*Isolates*	*Species*	*Shannon H'*	*Avg. Bootstrap*	*Dominant Genera*
Gram-negative	27	16	2.68	74%	Acinetobacter (25%)
Gram-positive	17	10	1.92	82%	Bacillus (29.4%)

Figure S3: **Taxonomic distribution and diversity indices of the isolated microbial community.** The 3D pie chart illustrates the relative abundance (percentage) and distribution of families and genera identified from meat-contact surfaces. Numerical values in parentheses indicate the absolute count of isolates per genus. The accompanying table summarizes the comparative diversity between Gram-negative and Gram-positive populations, featuring the Shannon-Wiener Diversity Index (H'), average bootstrap support from phylogenetic reconstruction, and the most prevalent genera within each group.

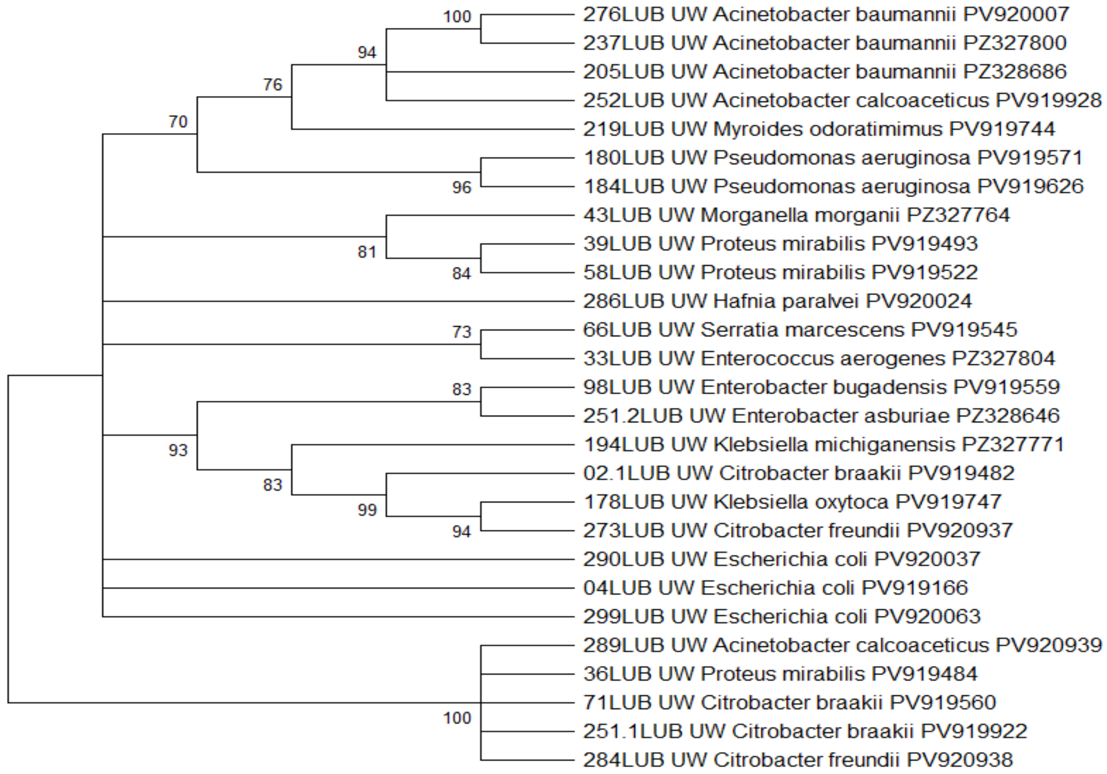

(A)

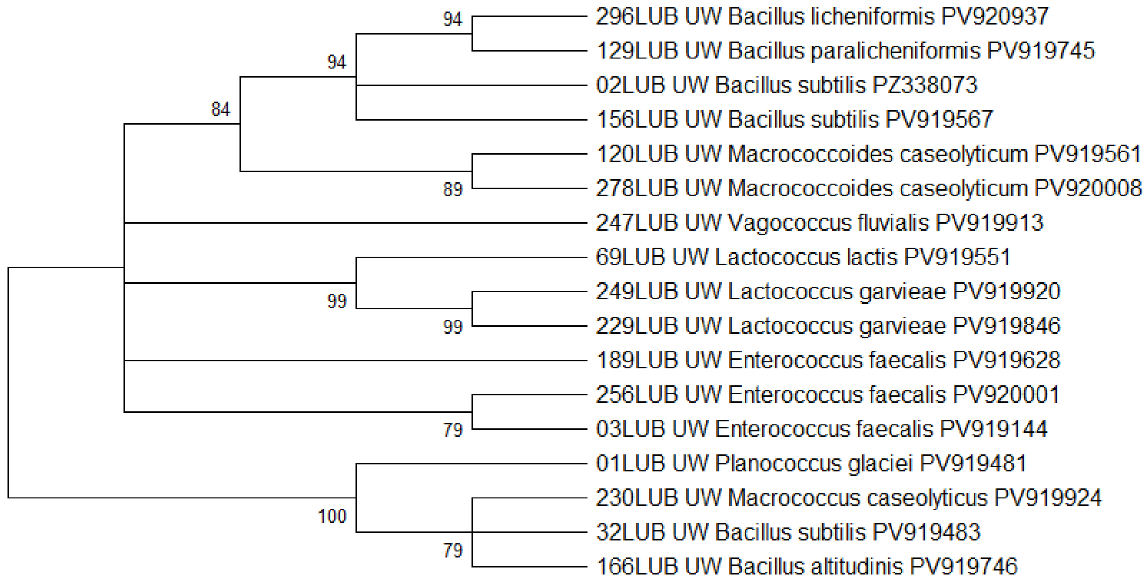

(B)

Figure S4: **Molecular phylogenetic characterization of the bacterial community isolated from meat-contact surfaces.** Evolutionary relationships were reconstructed using the Neighbor-Joining method in MEGA 11, based on 16S rRNA gene sequences. The percentage of replicate trees in which the associated

taxa clustered together in the bootstrap test (1,000 replicates) is shown next to the branches; nodes with values <70% are not indicated to prioritize statistically robust clades. Terminal leaf labels include the isolate ID, taxonomic assignment, and the corresponding NCBI GenBank accession number. **(A)** Phylogenetic tree of Gram-negative isolates, demonstrating the prevalence of Enterobacteriaceae and Moraxellaceae clades. **(B)** Phylogenetic tree of Gram-positive isolates, highlighting the dominance of Bacillaceae and Enterococcaceae.

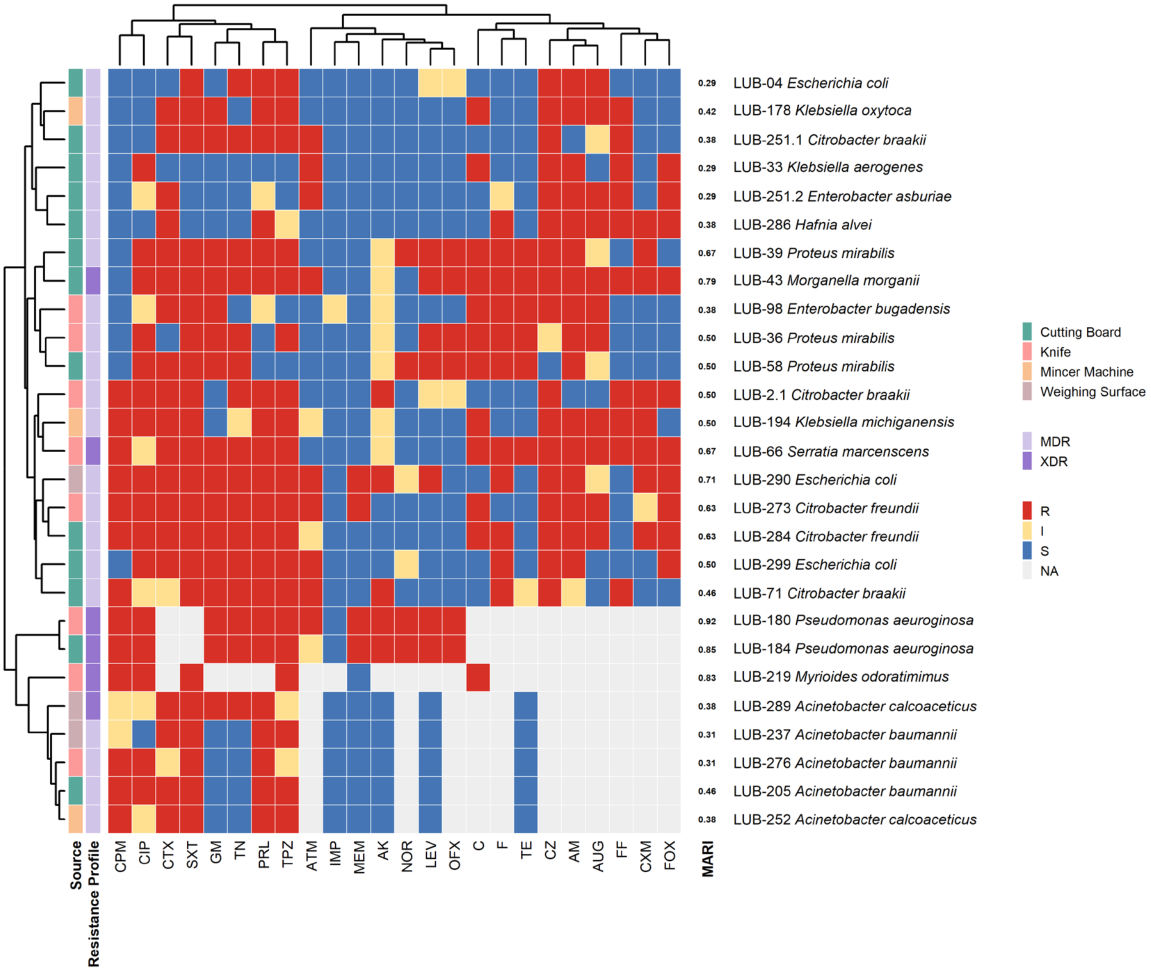

Figure S5: **Spatial heat grid analysis of Gram-negative isolates based on antimicrobial susceptibility profiling using the Disc Diffusion method.** Hierarchical clustering of phenotypic resistance patterns is integrated with metadata regarding isolate source and resistance categorization. The heatmap highlights the distribution of Multidrug-Resistant (MDR) and Extensively Drug-Resistant (XDR) clusters across different meat-processing contamination hotspots. The Multiple Antibiotic Resistance Index (MARI) for each isolate is provided along the right-hand margin, indicating the level of resistance. Dendrograms represent the proximity of resistance phenotypes (Y-axis) and the relatedness of antimicrobial efficacy (X-axis).

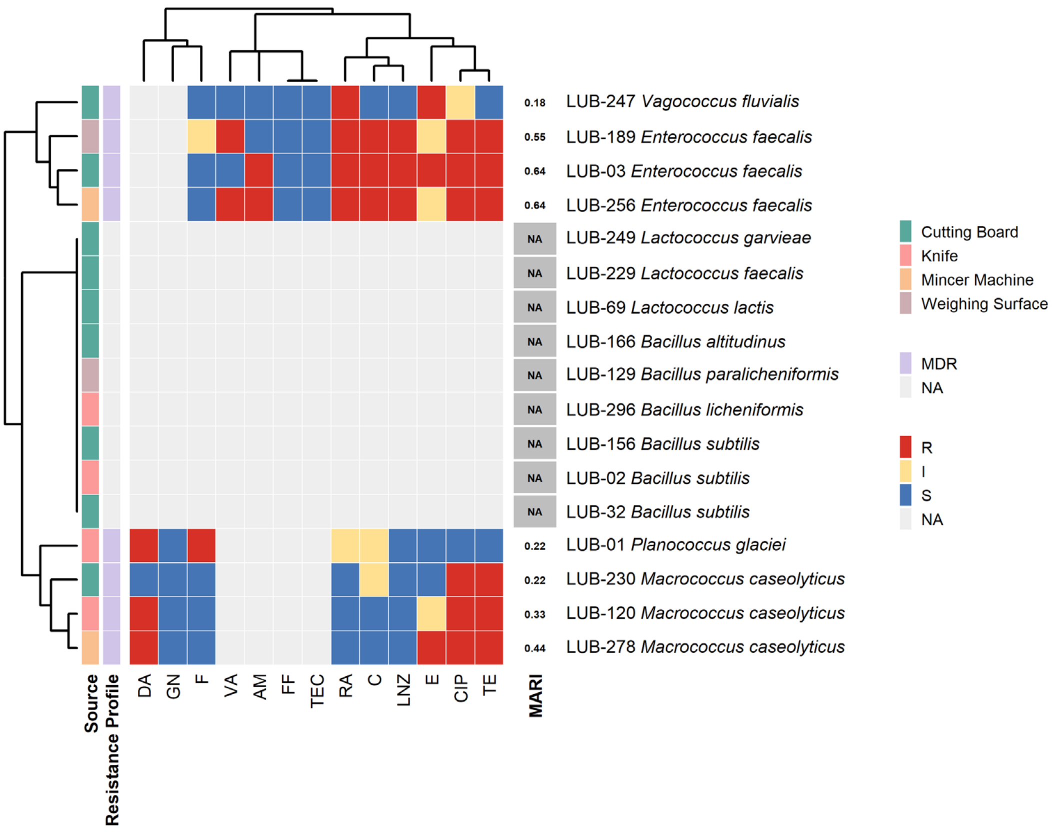

Figure S6: **Antibiogram heatmap and hierarchical clustering of Gram-positive isolates using disc diffusion assay.** The spatial heat grid illustrates the antimicrobial susceptibility profiles of 17 Gram-

positive isolates against a panel of clinically relevant antibiotics. Colors indicate phenotypic responses: Red (R, Resistant), Yellow (I, Intermediate), Blue (S, Susceptible), and Grey (NA, Not Applicable/Not Tested). Vertical dendrograms group isolates based on phenotypic similarity, revealing high-resistance clusters among *Enterococcus faecalis* and *Macrococcus caseolyticus*.
